# Supplementary material for: Community Health Impacts of the Trident Copper Mine Project in Northwestern Zambia: Results from Repeated Cross-Sectional Surveys
Source: Int J Environ Res Public Health. 2020 May 21;17(10):3633. doi: 10.3390/ijerph17103633 (PMC7277077; doi:10.3390/ijerph17103633)
Supplement: Supplementary file 1 [file ijerph-17-03633-s001.pdf]

Supplementary Material

Community Health Impacts of the Trident Copper Mine Project in Northwestern Zambia: Results from Repeated Cross-Sectional Surveys

Astrid M. Knoblauch [1,2,*], Andrea Farnham [1,2], Hyacinthe R. Zabré [1,2], Milka Owuor [3], Colleen Archer [4], Kennedy Nduna [5], Marcus Chisanga [6], Leonard Zulu [7], Gertrude Musunka [6], Jürg Utzinger [1,2], Mark J. Divall [3], Günther Fink [1,2] and Mirko S. Winkler [1,2]

1 Swiss Tropical and Public Health Institute, P.O. Box, 4002 Basel, Switzerland; andrea.farnham@swisstph.ch (A.F.); raogohyacinthe.zabre@swisstph.ch (H.R.Z.); juerg.utzinger@swisstph.ch (J.U.); guenther.fink@swisstph.ch (G.F.); mirko.winkler@swisstph.ch (M.S.W.)

2 University of Basel, P.O. Box, 4001 Basel, Switzerland

3 Shape Consulting, P.O. Box 602, St Peter Port GY1, Guernsey, UK; mowuor@shapeconsulting.org (M.O.); mdivall@shapeconsulting.org (M.J.D.)

4 Pollution Research Group, Department of Engineering, University of KwaZulu-Natal, 4041 Durban, South Africa; archerc@ukzn.ac.za

5 Nvumabaranda Public Health Services, Ndola, Zambia; kennedy.nduna@yahoo.com

6 First Quantum Minerals Limited, Lusaka, Zambia; marcus.chisanga@fqml.com (M.C.); gertrude.musunka@fqml.com (G.M.)

7 Independent researcher, Lusaka, Zambia; leonardzulu@gmail.com

* Correspondence: astrid.knoblauch@swisstph.ch

Received: 23 April 2020; Accepted: 19 May 2020; Published: date

Table S1. Vector control and *Plasmodium falciparum* parasitaemia indicators, Trident project, Zambia (2011, 2015, 2019).

	n	2011 BHS (%; 95% CI)	n	2015 FUHS (%; 95% CI)	n	2019 FUHS (%; 95% CI)	Interaction between Year (2011 *vs*. 2019) and Impact Status	
							OR (95% CI)	*p*-Value
**Households With at Least One Bednet**							0.58 (0.36–1.06)	0.080
Total impacted	251	35.1 (29.1–41.3)	353	80.7 (76.2–84.7)	351	72.4 (67.4–77.0)		
Total comparison	29	24.1 (10.3–43.5)	128	77.3 (69.1–84.3)	158	68.4 (60.5–75.5)		
Total overall	280	33.9 (28.4–39.8)	481	79.8 (76.0–83.3)	509	71.1 (67.0–75.0)		
**Children 6–59 Months Sleeping Under a Bednet**							1.05 (0.52–2.09)	0.902
Total impacted	415	28.0 (23.7–32.5)	534	60.5 (56.2–64.7)	566	52.5 (48.3–56.7)		
Total comparison	42	28.6 (15.7–44.6)	237	56.5 (50.0–62.9)	268	52.6 (46.4–58.7)		
Total overall	457	28.0 (23.9–32.4)	771	59.3 (55.7–62.8)	834	52.5 (49.1–56.0)		
**Households Sprayed in the 12 Months Preceding the Survey**							no data for 2011	
Total impacted		n/m	354	69.8 (64.7–74.5)	351	81.8 (77.3–85.7)		
Total comparison		n/m	128	28.9 (21.1–37.6)	158	88.6 (82.6–93.1)		
Total overall		n/m	482	58.9 (54.4–63.4)	509	83.9 (80.4–87.0)		
**Prevalence of *Plasmodium falciparum* malaria in children 6-59 months**							1.18 (0.55–2.50)	0.672
Total impacted	418	17.5 (13.9–21.5)	544	30.7 (26.8–34.8)	566	39.8 (35.7–43.9)		
Total comparison	41	34.1 (20.0–50.6)	237	55.3 (48.7–61.8)	268	57.8 (51.7–63.8)		
Total overall	459	19.0 (15.5–22.8)	781	38.2 (34.7–41.7)	834	45.6 (42.1–49.0)		

BHS, baseline health survey; CI, confidence interval; FUHS, follow-up health survey; n/m, not measured

Table S2. Nutritional indicators and anaemia, Trident project, Zambia (2011, 2015, 2019).

	n	2011 BHS (%; 95% CI)	n	2015 FUHS (%; 95% CI)	n	2019 FUHS (%; 95% CI)	Interaction Between Year (2011 *vs*. 2019) and Impact Status	
							OR (95% CI)	*p*-Value
**Prevalence of Wasting (Low Weight-for-height) in Children 0–59 Months**							0.59 (0.06–6.06)	0.650
Total impacted	436	1.8 (0.8–3.6)	633	0.8 (0.3–1.8)	609	1.3 (0.6–2.6)		
Total comparison	51	2.0 (0.1–10.4)	256	0.8 (0.1–2.8)	295	2.4 (1.0–4.8)		
Total overall	487	1.8 (0.8–3.5)	889	0.8 (0.3–1.6)	904	1.7 (1.0–2.7)		
**Prevalence of Stunting (Low Height-for-age) in Children 0–59 Months**							0.52 (0.27–1.01)	0.054
Total impacted	436	49.5 (44.8–54.3)	633	36.7 (32.9–40.5)	609	27.9 (24.4–31.7)		
Total comparison	51	41.2 (27.6–55.8)	265	46.3 (40.7–52.0)	295	34.6 (29.2–40.3)		
Total overall	487	48.7 (44.1–53.2)	889	39.9 (36.7–43.1)	904	30.0 (27.1–33.2)		
**Prevalence of Underweight (Low Weight-for-age) in Children 0–59 Months**							0.39 (0.12–1.22)	0.106
Total impacted	436	13.1 (10.1–16.6)	633	7.6 (5.6–9.9)	609	8.7 (6.6–11.2)		
Total comparison	51	7.8 (2.2–18.9)	256	10.8 (7.6–14.8)	295	12.2 (8.7–16.5)		
Total overall	487	12.5 (9.7–15.8)	889	8.6 (6.9–10.6)	904	9.8 (8.0–12.0)		
**Prevalence of Anaemia (Haemoglobin <11 g/dl) in Children 6–59 Months**							1.5 (0.73–3.09)	0.268
Total impacted	418	46.4 (41.6–51.3)	547	40.6 (36.5–44.9)	566	44.2 (40.0–48.4)		
Total comparison	42	64.3 (48.0–78.4)	237	48.9 (42.4–55.5)	268	52.2 (46.1–58.4)		
Total overall	460	48.0 (43.4–52.7)	781	43.1 (39.6–46.7)	834	46.8 (43.3–50.2)		

BHS, baseline health survey; CI, confidence interval; FUHS, follow-up health survey

Table S3. Schistosomiasis and soil-transmitted helminths indicators in school attendees aged 9-14 years, Trident project, Zambia (2011, 2015, 2019).

	n	2011 BHS (%; 95% CI)	n	2015 FUHS (%; 95% CI)	n	2019 FUHS (%; 95% CI)	Interaction between year (2011 *vs.* 2019) and impact status	
							OR (95% CI)	p-value
**Prevalence of *Schistosomiasis haematobium***							2.28 (0.88-5.91)	0.091
Total impacted	278	19.4 (14.9–24.8)	328	11.9 (8.6–15.9)	330	10.0 (7.0–13.8)		
Total comparison	34	73.5 (55.6–87.1)	120	35.0 (26.5–44.2)	150	36.0 (28.3–44.2)		
Total overall	312	25.3 (20.8–30.5)	448	18.1 (14.6–22.0)	480	18.1 (14.8–21.9)		
**Prevalence of *Schistosomiasis mansoni***							Interaction model was unable to be adjusted for year and impact due to 0.0% prevalence.	
Total impacted	276	1.1 (0.2–3.1)	328	8.2 (5.5–11.8)	330	7.6 (5.0–11.0)		
Total comparison	34	0.0	120	0.8 (0.0–4.6)	150	0.0 (-)		
Total overall	310	1.0 (0.2–2.8)	448	6.3 (4.2–8.9)	480	5.2 (3.4–7.6)		
**Prevalence of *Ascaris lumbricoides***							Interaction model was unable to be adjusted for year and impact due to 0.0% prevalence.	
Total impacted	276	0.7 (0.0–2.6)	328	1.8 (0.7–3.9)	330	0.0		
Total comparison	34	0.0	120	0.0	150	0.0		
Total overall	310	0.6 (0.0–2.3)	448	1.3 (0.5–2.9)	480	0.0		
**Prevalence of *Trichuris trichiura***							Interaction model was unable to be adjusted for year and impact due to 0.0% prevalence.	
Total impacted	276	8.3 (5.4–12.2)	328	6.7 (4.3–10.0)	330	3.0 (1.5–5.5)		
Total comparison	34	0.0	120	10.8 (5.9–17.8)	150	8.7 (4.7–14.4)		
Total overall	310	7.4 (4.8–10.9)	448	7.8 (5.5–10.7)	480	4.8 (3.1–7.1)		
**Prevalence of *hookworm***							0.27 (0.11-0.64)	0.003
Total impacted	276	62.7 (56.7–68.4)	328	56.1 (50.5–61.5)	330	13.0 (9.6–17.1)		
Total comparison	34	58.8 (40.7–75.4)	120	73.3 (64.5–81.0)	150	32.0 (24.6–40.1)		
Total overall	310	62.3 (56.6–67.7)	448	60.7 (56.0–65.3)	480	19.0 (15.5–22.8)		
**Proportion of Children Without Any Parasites**							0.84 (0.27-2.66)	0.771
Total impacted	276	29.4 (24.1–35.1)	328	35.7 (30.5–41.1)	330	70.3 (65.1–75.2)		
Total comparison	34	11.8 (3.3–27.5)	120	14.2 (8.5–21.7)	150	47.3 (39.1–55.6)		
Total overall	310	27.5 (22.6–32.8)	448	29.9 (25.7–34.4)	480	63.1 (58.6–67.5)		

BHS, baseline health survey; CI, confidence interval; FUHS, follow-up health survey

Table S4. Women's health indicators, Trident project, Zambia (2011, 2015, 2019).

	n	2011 BHS (%; 95% CI)	n	2015 FUHS (%; 95% CI)	n	2019 FUHS (%; 95% CI)	Interaction between year (2011 *vs.* 2019) and impact status	
							OR (95% CI)	*p*-Value
**Prevalence of Mothers 15–49 Years that Delivered the Last Born Child in a Health Facility**							0.46 (0.15–1.43)	0.178
Total impacted	282	64.9 (59.1–70.4)	432	83.0 (79.1–86.4)	416	95.0 (92.4–96.8)		
Total comparison	31	54.8 (36.0–72.7)	148	78.4 (70.9-84.7)	185	96.2 (92.4–98.5)		
Total overall	319	63.9 (58.4–69.2)	583	81.8 (78.4-84.9)	601	95.3 (93.3–96.9)		
**Prevalence of Underweight in Women 15–49 Years**							no data for 2011	
Total impacted		n/m	429	7.9 (5.6–10.9)	416	9.9 (7.2–13.1)		
Total comparison		n/m	148	15.5 (10.1–22.4)	185	11.9 (7.6–17.4)		
Total overall		n/m	577	9.9 (7.6–12.6)	601	10.5 (8.1–13.2)		
**Prevalence of Anaemia in Women 15–49 Years (Hb <11 g/dl Pregnant; <12 g/dl Non-pregnant)**							1.42 (0.54–3.75)	0.478
Total impacted	288	17.0 (12.9–21.9)	432	25.2 (21.2–29.6)	416	16.8 (13.6–20.8)		
Total comparison	31	25.8 (11.8–44.6)	148	24.3 (17.7–32.1)	185	19.5 (14.0–25.9)		
Total overall	319	17.9 (13.8–22.5)	580	25.0 (21.5–28.7)	601	17.6 (14.7–20.9)		
**Prevalence of syphilis in women 15–49 years**							no data for 2011	
Total impacted		n/m	411	4.4 (2.6–6.8)	416	5.3 (3.3–7.9)		
Total comparison		n/m	131	3.8 (1.6–8.7)	185	3.8 (1.5–7.6)		
Total overall		n/m	542	4.2 (2.7–6.3)	601	4.8 (3.3–6.9)		

BHS, baseline health survey; CI, confidence interval; FUHS, follow-up health survey; Hb, haemoglobin; n/m, not measured
